# *Allophoma* species (Pleosporales: Didymellaceae) associated with *Thunbergia grandiflora* in Guangxi Province, China

**DOI:** 10.3897/BDJ.9.e63643

**Published:** 2021-03-01

**Authors:** Jun Yuan, Xiang-Yu Zeng, Kun Geng, Nalin N. Wijayawardene, Jayarama D. Bhat, Shi-Ping Wu, Yong Wang, Zai-Fu Yang

**Affiliations:** 1 Department of Plant Pathology, Agricultural College, Guizhou University, Guiyang, China Department of Plant Pathology, Agricultural College, Guizhou University Guiyang China; 2 Guiyang plant protection and inspection station, Guiyang, China Guiyang plant protection and inspection station Guiyang China; 3 Center for Yunnan Plateau Biological Resources Protection and Utilization, Qujing Normal University, Qujing, China Center for Yunnan Plateau Biological Resources Protection and Utilization, Qujing Normal University Qujing China; 4 No. 128/1-J, Azad Co-Op Housing Society, Curca P.O, Goa, India No. 128/1-J, Azad Co-Op Housing Society, Curca P.O Goa India; 5 The Institute of Plant Protection, Guizhou Academy of Agricultural Sciences, Guiyang, China The Institute of Plant Protection, Guizhou Academy of Agricultural Sciences Guiyang China

**Keywords:** one new species, Didymellaceae, phylogeny, taxonomy

## Abstract

**Background:**

*Thunbergia grandiflora* belongs to the family Acanthaceae and is a widely distributed dicotyledonous plant in tropical and subtropical regions. Three isolates of *Allophoma* (Dothideomycetes, Pleosporales, Didymellaceae) were collected from leaves of *T. grandiflora* in Guangxi Province, China.

**New information:**

Phylogenetic analyses of a combined ITS–LSU–*rpb2*–*tub2* dataset indicate that one of our three strains represents an undescribed species with close affinity to *A. minor* and the other two strains clustered amongst other isolates of *A. pterospermicola*. Evidence from morphology and sequence analysis indicates that GUCC 2070.7 is a new species that we introduce here as *A. thunbergiae*. This is the first report about taxa of *Allophoma* from this host plant.

## Introduction

Didymellaceae was established by De Gruyter et al. (2009) with *Didymella* as the type genus. It is the largest family in the Pleosporales and accommodates more than 5400 taxon names (Crous et al. 2004), including saprobic, endophytic and pathogenic species (Aveskamp et al. 2008, Aveskamp et al. 2010, Marin-Felix et al. 2017). A great part of Didymellaceae species are reported as plant pathogens, which cause severe economic losses to many crops (Aveskamp et al. 2008). Recently, Didymellaceae was revised, based on morphological and phylogenetic analyses of ex-type sequences of LSU, ITS, *rpb2* and *tub2* loci, resulting in 19 genera (Chen et al. 2015, Chen et al. 2017), Currently, 37 genera are accepted (Wijayawardene et al. 2017, Wijayawardene et al. 2020, Valenzuela-Lopez et al. 2018, Hou et al. 2020a, Hou et al. 2020b, Phukhamsakda et al. 2020).

*Allophoma* is presently accepted with 14 species (Hongsanan et al. 2020, Hou et al. 2020a, Hyde et al. 2020, Wijayawardene et al. 2020) and two of them were firstly obtained from Guizhou and Guangxi Provinces, China (Chen et al. 2017, Marin-Felix et al. 2019). The genus includes several important plant-pathogenic taxa, for example, *Allophoma labilis* (basionym: *Phoma labilis*), which often cause leaf necrosis, canker and stem lesions or stem rot, resulting in a negative effect on the health of plants (Zimowska 2011, Garibaldi et al. 2012, Nagarjun and Suryanarayana 2016, O'Neill and Mayne 2016, Babaahmadi et al. 2018,Jayasiri et al. 2019). *Allophoma* is characterised by superficial or immersed pycnidial conidiomata with ostioles, a 2−5-layered pseudo-parenchymatous wall, phialidic conidiogenous cells and aseptate variously-shaped, mostly guttulate conidia. The size of the pycnidia, conidiogenous cells and conidia are used to distinguish amongst different species in *Allophoma* (Chen et al. 2015).

In recent years, most species of fungi have been described from Asia, mostly China (Cheek et al. 2020). Our research group investigates the fungi on medicinal plants in south-western China, which has, thus far, resulted in the discovery of several new taxa (Long et al. 2019, Zhang et al. 2020a, Zhang et al. 2020b, An et al. 2021). Here, we studied diseased leaves of *Thunbergia grandiflora* collected from the Medicinal Botanical Garden in Nanning City, Guangxi Province, China. Following isolation, purification, morphological examination and phylogenetic analyses, a new species and one known species were discovered.

## Materials and methods

### Isolation and morphological study

The samples were collected in 2017 at the Medicinal Botanical Garden, Nanning, Guangxi, China. Single spore isolates were obtained on oatmeal agar (OA), malt extract agar (MEA) and potato dextrose agar (PDA), followed by incubation at 25 °C. Colony diameters were measured after 1 week (Boerema et al. 2004). The colour of colonies of inoculated Petri dishes was determined following Rayner (1970). Morphological structures were examined and photographed using a Nikon Eclipse 80i microscope. Micro-morphological descriptions and measurements of mature conidiomata, conidia and conidiogenous cells on OA or MEA and PDA cultures were based on Aveskamp et al. (2010). The holotype specimen is deposited at the Herbarium of the Department of Plant Pathology, Agricultural College, Guizhou University (HGUP). An ex-type culture of the new taxon is deposited at the Culture Collection of the Department of Plant Pathology, Agriculture College, Guizhou University (GUCC) (Table 1).

### DNA isolation, PCR and sequencing

Fungal mycelia were scraped off the surface of the pure culture plate with a sterile scalpel. Total genomic DNA was extracted using the A BIOMIGA Fungus Genomic DNA Extraction Kit (GD2416, BIOMIGA, San Diego, California, USA). Four loci of each fungal strains were amplified, including the internal transcribed spacer (ITS) region with primers V9G (De Hoog and Van den Ended 1998) and ITS4 (White et al. 1990); the large subunit (LSU) of the ribosomal RNA gene with primers LR0R (Hopple 1994), LR5 and LR7 (Vilgalys and Hester 1990); the second-largest subunit of the RNA polymerase II (*rpb2*) wih primers RPB2-5F2 (Sung et al. 2007) and fRPB2-7cR (Liu et al. 1999); and β-tubulin (*tub2*) with primers Btub2Fd and Btub4Rd (Woudenberg et al. 2009). DNA amplifications were performed in 25-μl reaction volumes, containing 2.5 μl 10 × PCR buffer, 1 μl of each primer (10 μM), 1 μl template DNA, 0.25 μlTaq DNA polymerase (Promega, Madison, WI, USA) and 18.5 μl ddH_2_O. The PCR cycling conditions for ITS were as follows: initial denaturation at 95°C for 5 min; then 35 cycles of denaturation at 95°C for 30 s, annealing at 52°C for 45 s and extension at 72°C for 90 s; and final extension at 72°C for 10 min. For LSU: initial denaturation at 98°C for 3 min; then 35 cycles of denaturation at 98°C for 30 s, annealing at 45°C for 27 s and extension at 72°C for 30 s; and final extension at 72°C for 10 min. For *rpb2*: initial denaturation at 95°C for 5 min; then 40 cycles of denaturation at 95°C for 1 min, annealing at 55°C for 2 min and extension at 72°C for 90 s; and final extension at 72°C for 10 min. For *tub2*: initial denaturation at 94°C for 3 min; then 35 cycles of denaturation at 94°C for 1 min, annealing at 58°C for 45 s and extension at 72°C for 1 min; and final extension at 72°C for 10 min. The amplification products were sent to SinoGenoMax (Beijing) for sequencing. The newly-generated DNA sequences were submitted to GenBank (accession numbers in Table 1). The DNA base differences on four loci amongst our strains and ex-type or representative strains of relative *Allophoma* taxa are shown in Table 2.

### Sequence alignment and phylogenetic analyses

The related DNA sequences for phylogenetic analyses in this study were downloaded from GenBank (Table 1). Amongst them, *Stagonosporopsis loticola* (CBS 562.81) is regarded as outgroup taxon. Alignments for four individual loci were constructed (ITS, *rpb2*, *tub2* and LSU) in MAFFT v7.307 online version (Katoh and Standley 2016) and were manually edited in MEGA v. 6.0 when necessary (Tamura et al. 2013). The concatenated aligned dataset and each locus were analyzed separately using Maximum Likelihood (ML), Bayesian Inference (BI) and Maximum Parsimony (MP).The best fit substitution model for each gene was tested from eleven substitution schemes by using ‘jModelTest2 on XSEDE’ tool (Darriba et al. 2012) at the CIPRES web portal (Miller et al. 2010), and determined by the Bayesian information criterion (BIC).ML analysis was performed using RAxML-HPC2 v. 8.2.12 (Stamatakis 2014) as implemented on the CIPRES Science Gateway, with the GTR+G+I model and 1,000 rapid bootstrap (BS) replicates for four genes. For BI analysis, the best substitution model for each partition was determined with the program MrModeltest 2.2 (Nylander 2004) to be GTR+G+I. BI analysis was performed using MrBayes v.3.2.6 (Ronquist et al. 2012) as implemented on the Cipres portal (Miller et al. 2010). Parameters and tree samples were summarized with a burn-in fraction of 0.25, which were checked against the log likelihood by sampled generation plot. MP analysis was performed in PAUP v. 4.0b10 (Swofford 2002) using the heuristic search option with 1,000 random taxa additions and tree bisection and reconnection (TBR) as the branch-swapping algorithm. The maxtrees were set as 5000 to build up the phylogenetic tree. The Tree Length (TL), Consistency Indices (CI), Retention Indices (RI), Rescaled Consistency Indices (RC) and Homoplasy Index (HI) were calculated for each tree generated.

## Taxon treatments

### Allophoma thunbergiae

Jun Yuan & Yong Wang bis sp. nov.

12E84C9E-A66C-5636-946A-8241BBD00913

558130

#### Materials

- **Type status:** Holotype. **Occurrence:** recordedBy: Jun Yuan; occurrenceID: GUCC 2070.7; **Taxon:** scientificName: Allophoma thunbergiae; order: Pleosporales; family: Didymellaceae; genus: Allophoma; **Location:** country: China; stateProvince: GuangXi; locality: Nanning City, Guangxi Medicinal Botanical Garden; verbatimCoordinates: 22°51’N, 108°19’E; **Identification:** identifiedBy: Jun Yuan; dateIdentified: 2020; **Record Level:** collectionID: HGUP 2070.7

#### Description

**Figure 1.**
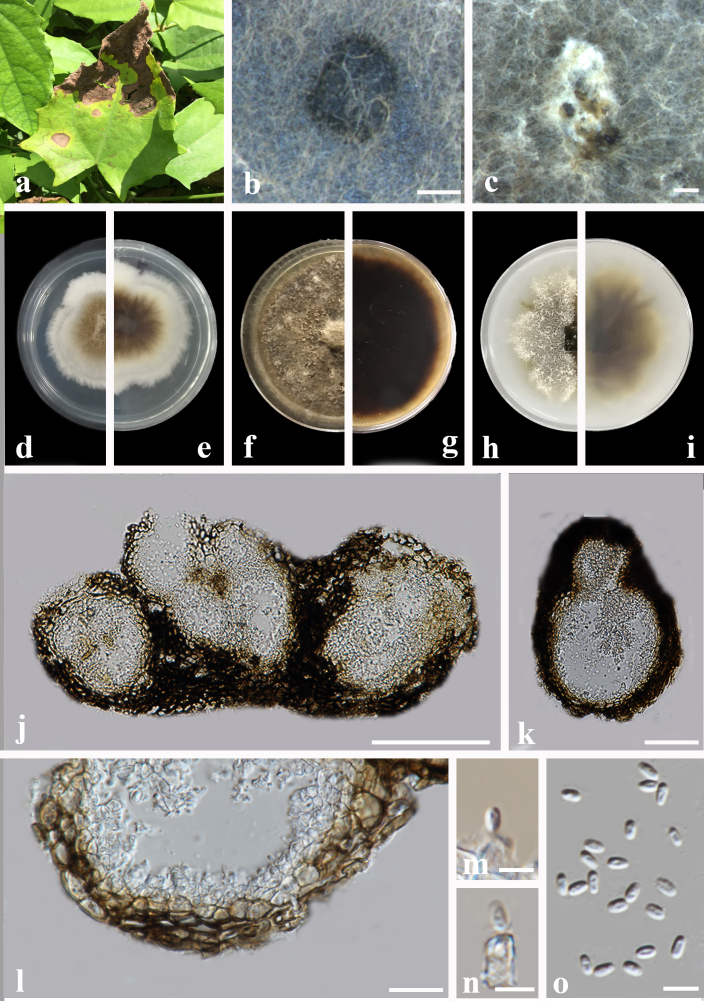
***Allophoma thunbergiae*** (GUCC2070.7) **a.** Leaf symptoms on the host; **b**, **c.** Pycnidia forming on PDA; **d**, **e.** Colony on PDA (front and reverse); **f**, **g.** Colony on MEA (front and reverse); **h**, **i.** Colony on OA (front and reverse); **j**, **k.** Section of pycnidium; **l.** Section of pycnidial wall; **m**, **n.** Conidiogenous cells; **o.** Conidia. Scale bars: b, c = 500 μm; j = 100 μm; k = 50 μm; l = 20 μm; m−o = 5 μm.

Pathogenic on the leaf spot of *Thunbergia grandiflora*. Lesions initially on the upper leaf surface, scattered, distinct, irregular, the maximum length of the spot more than 10-15 mm, the edge of the spots yellow, the centre of necrotic section brown, on the lower leaf surface similar. Sexual morph: Undetermined. Asexual morph (Fig. 1): Coelomycetous. Conidiomata pycnidial, mostly solitary or aggregated, subglobose to irregular, dark brown, glabrous, covered with some hyphal outgrowths, produced on the agar surface or (semi-)immersed, ostiolate, (39−)44−200 × (48−)49−230 μm (x̄ = 108.9 × 138.9 μm, n = 20). Ostioles 1−3, with a short neck, slightly papillate or sometimes non-papillate. Pycnidial wall pseudoparenchymatous, composed of oblong to isodiametric cells, 3−4 layered, 14−32 μm thick (x̄ = 20.8 μm, n = 10). Conidiogenous cells phialidic, hyaline, smooth, ampulliform to doliiform, 4.5−7 × 4−5 μm (x̄ = 4.9 × 4.6 μm, n = 10), with a distinct periclinal thickening. Conidia oblong to cylindrical, slightly obovoid, smooth and thin-walled, hyaline, aseptate, 3−5 × 1.5−2.5 μm (x̄ = 3.6 × 2.2 μm, n = 20), with two minutes guttules. Conidial exudates not recorded.

Culture characteristics: Colonies on PDA, 46−57 mm diameter after 1 week, irregular at margin, aerial mycelia floccose, grey with a white margin, brown near the centre; reverse pale brown, with a white margin. Colonies on MEA 44−47 mm diameter after 1 week, regular at margin, covered by brown, dense aerial mycelia, yellow near the centre; reverse greyish-brown. Colonies on OA, 41−46 mm diameter after 1 week, irregular at margin, covered by white aerial mycelia sparse, brownish, reverse buff to yellowish-olivaceous.

#### Etymology

In reference to the host (*Thunbergia grandiflora*), from which the fungus was isolated.

### Allophoma pterospermicola

Qian Chen & L. Cai, Stud. Mycol. 94: 4 (2019)

D929DD22-05E0-57B7-871B-E45F483E537B

828313

#### Materials

- **Type status:** Other material. **Occurrence:** recordedBy: Jun Yuan; occurrenceID: GUCC 2070.3 and GUCC 2070.6; **Taxon:** scientificName: Allophoma pterospermicola; order: Pleosporales; family: Didymellaceae; genus: Allophoma; **Location:** country: China; stateProvince: GuangXi; locality: Nanning City, Guangxi Medicinal Botanical Garden; verbatimCoordinates: 22°51’N, 108°19’E; **Identification:** identifiedBy: Jun Yuan; dateIdentified: 2020; **Record Level:** collectionID: HGUP 2070.3 and HGUP 2070.6

#### Description

**Figure 2.**
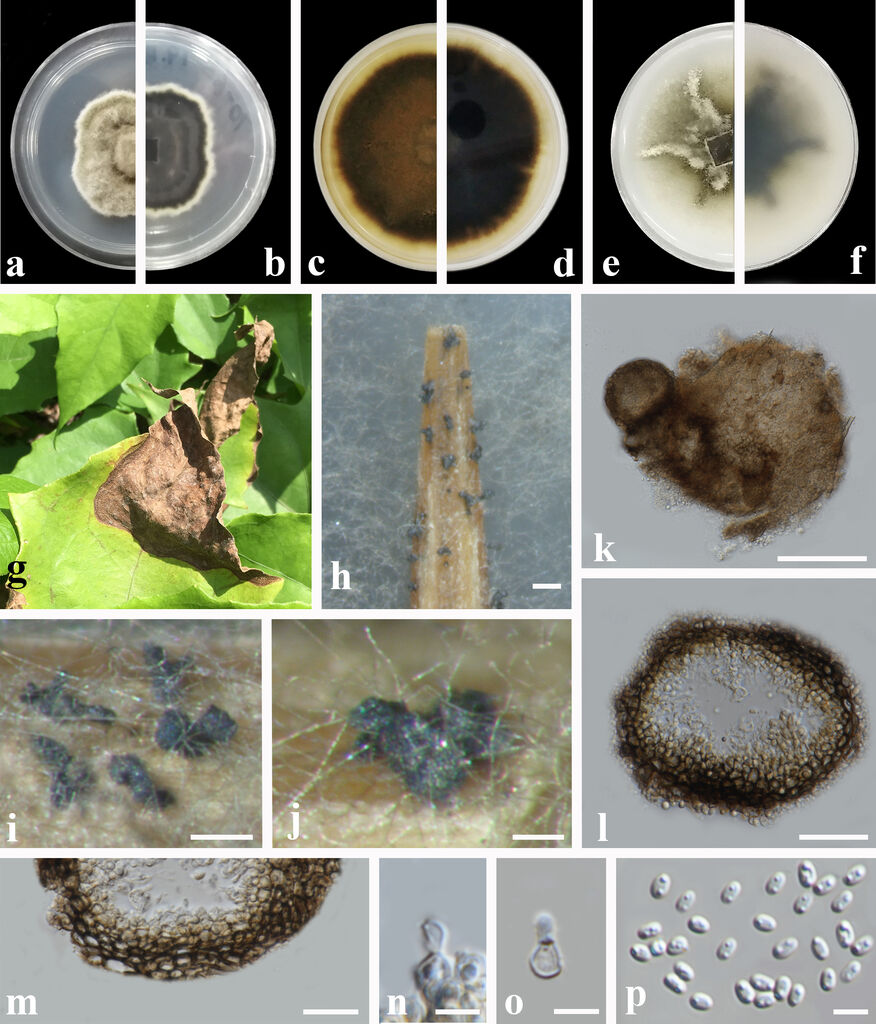
***Allophoma pterospermicola*** (GUCC2070.3) **a**, **b.** Colony on PDA (front and reverse); **c**, **d.** Colony on MEA (front and reverse); **e**, **f.** Colony on OA (front and reverse); **g.** Leaf symptoms on the host; **h**-**j.** Pycnidia forming on the toothpick; **k.** Pycnidium; **l.** section of pycnidium; **m.** Section of pycnidial wall; **n**, **o.** Conidiogenous cells; **p.** Conidia. Scale bars: h, i = 500 μm; j = 100 μm; k = 50 μm; l, m = 20 μm; n−p = 5 μm.

Pathogenic on the leaf spot of *Thunbergia grandiflora*. Lesions initially on the upper leaf surface, scattered, distinct, irregular, the maximum length of the spot more than 10-13 mm, the edge of the spots yellow, the necrotic section brown at the later stage connected to form the dead leaves, on the lower leaf surface similar. Sexual morph: Undetermined. Asexual morph (Fig. 2): Coelomycetous. Conidiomata pycnidial, mostly aggregated and those aggregates are solitary, scattered, globose, subglobose or sometimes irregular, dark brown, glabrous, covered with some hyphal outgrowths, produced on the toothpick surface, ostiolate, (42−)52−208 × (25−)63−147 μm (x̄ = 108.1 × 99.3 μm, n = 20). Ostiole single, with a short neck, slightly papillate. Pycnidial wall pseudoparenchymatous, composed of oblong to isodiametric cells, 3−6 layers, 18−36 μm thick (x̄ = 23.6 μm, n = 10). Conidiogenous cells phialidic, hyaline, smooth, ampulliform to doliiform, 3.5−6 × 3.5−4 μm (x̄ = 3.8 × 4.3 μm, n = 10). Conidia ellipsoidal to oblong, incidentally slightly obovoid, smooth and thin-walled, hyaline, aseptate, 2.5−4 × 1.5−2.5 μm (x̄ = 3.5 × 2.5 μm, n = 20), with 2 distinct polar guttules. Conidial exudates not recorded.

Culture characteristics: Colonies on PDA, 46−50 mm diameter after 1 week, regular at margin, densely covered by floccose aerial mycelia, grey, with a white concentric ring near the margin; reverse pale black, with a white concentric ring near the margin. Colonies on MEA, 52−58 mm diameter after 1 week, regular at margin, dull green, aerial mycelia floccose, aerial mycelia sparsely, grey near the centre; reverse changing towards margin from the centre greyish-brown to brown. Colonies on OA 34−47 mm diameter after 1 week, irregular at margin, covered by floccose aerial mycelia, mycelia sparse in some furrowed zone, reverse buff to pale olivaceous.

## Analysis

**Figure 3.**
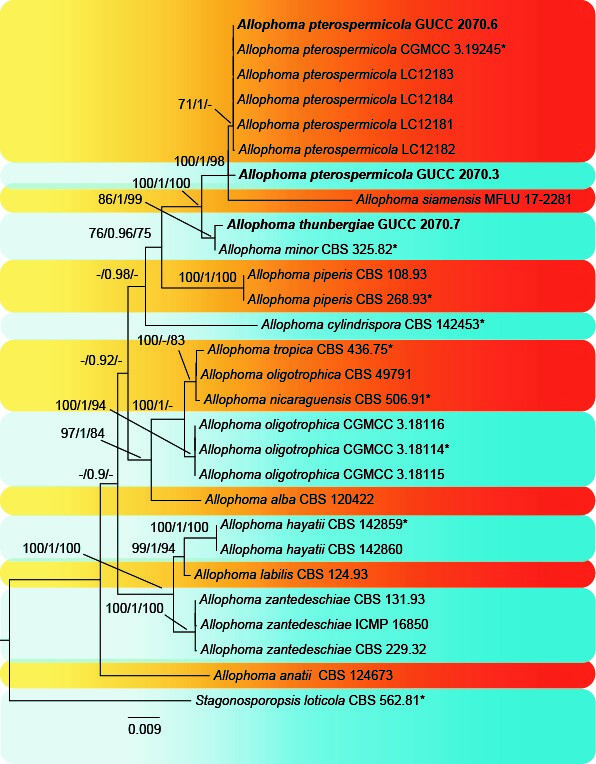
Phylogenetic tree inferred from a Maximum Parsimonious analysis, based on a concatenated alignment of ITS, *rpb2*, *tub2* and LSU sequences. ML bootstrap support values (MLBS) ≥ 70, BI posterior probabilities (BIPP) ≥ 0.90, and MP boostrap support values (MPBS) ≥ 70 are given at the nodes. The tree was rooted to *Stagonosporopsis loticola* (CBS 562.81). Newly-generated isolates are in bold. Ex-type strains are marked by an asterisk (*).

Phylogenetic analyses (Fig. 3

## Discussion

*Phoma* *sensu lato* was previously a large genus with *phoma-like* species (De Gruyter et al. 2012), but was recently characterised using molecular data, resulting in many species that were transferred to new genera, such as *Allophoma* (Chen et al. 2015). In this study, our isolates from *Thunbergia* (GUCC 2070.3, GUCC 2070.6 and GUCC 2070.7) represent species of *Allophoma* (Didymellaceae). One of these isolates, GUCC 2070.7, was retrieved close to *A. minor* in our phylogenetic tree (Fig. 3). In Table 3, we provide a comprehensive comparison of pycnidia, conidiogenous cells and conidia, which indicates that strain GUCC 2070.7 has smaller pycnidia (39−200 × 48−230 μm vs. 150−280 × 150−220 μm) and larger conidiogenous cells (4.5−7 × 4−5 μm vs. 4−5.5 × 3−4.5 μm) than *A. minor*. The phylogenetic analyses and comparison of DNA base pairs confirm that strain GUCC 2070.7 is different from *A. minor* sensu Jeewon and Hyde (2016). In summary, strain GUCC 2070.7 represented an undescribed species, *A. thunbergiae*, whereas strains GUCC 2070.3 and GUCC 2070.6 are *A. pterospermicola*, based on phylogenetic analyses and morphological studies (Chen et al. 2017, Fig. 3, Table 2). *Thunbergia grandiflora*, native to China, is here reported as a host for *Allophoma* species for the first time.

## Supplementary Material

XML Treatment for Allophoma thunbergiae

XML Treatment for Allophoma pterospermicola

## Figures and Tables

**Table 1.** Sequences that were used for phylogenetic analysis. The accession numbers in bold are those generated in this study. Ex-type strains are marked by an asterisk (*).

**Species**	**Strain number**	**GenBank accession numbers**			
		**LSU**	**ITS**	***rpb2***	***tub2***
*Allophoma alba*	CBS 120422	MN943671	MN973469	MT018044	MT005568
*A. anatii*	CBS 124673	MN943674	MN973472	MT018048	MT005571
*A. cylindrispora*	CBS 142453*	LN907376	LT592920	LT593058	LT592989
*A. hayatii*	CBS 142859	KY684814	KY684812	MF095108	KY684816
*A. hayatii*	CBS 142860	KY684815	KY684813	MF095109	KY684817
*A. labilis*	CBS 124.93	GU238091	GU237765	KT389552	GU237619
*A. minor*	CBS 325.82*	GU238107	GU237831	KT389553	GU237632
*A. nicaraguensis*	CBS 506.91*	GU238058	GU237876	KT389551	GU237596
*A. oligotrophica*	CBS 497.91	GU238059	GU237870	GU237597	LT623247
*A. oligotrophica*	CGMCC 3.18114*	KY742194	KY742040	KY742128	KY742282
*A. oligotrophica*	CGMCC 3.18115	KY742195	KY742041	KY742129	KY742283
*A. oligotrophica*	CGMCC 3.18116	KY742196	KY742042	KY742130	KY742284
*A. piperis*	CBS 268.93*	GU238129	GU237816	KT389554	GU237644
*A. piperis*	CBS 108.93	GU238130	GU237921	KT389555	GU237645
*A. pterospermicola*	CGMCC 3.19245*	MK088580	MK088573	MK088587	MK088594
*A. pterospermicola*	LC12181	MK088576	MK088569	MK088583	MK088590
*A. pterospermicola*	LC12182	MK088577	MK088570	MK088584	MK088591
*A. pterospermicola*	LC12183	MK088578	MK088571	MK088585	MK088592
*A. pterospermicola*	LC12184	MK088579	MK088572	MK088586	MK088593
*A. pterospermicola*	GUCC2070.6	**MW040200**	**MW036297**	**MW116818**	**MW116822**
*A. pterospermicola*	GUCC2070.3	**MW040199**	**MW036296**	**MW116817**	**MW116821**
*A. siamensis*	MFLU 17-2281	MK347959	MK347742	MK434912	MK412867
*A. thunbergiae*	GUCC2070.7	**MW040201**	**MW036298**	**MW116819**	**MW116823**
*A. tropica*	CBS 436.75*	GU238149	GU237864	KT389556	GU237663
*A. zantedeschiae*	CBS 131.93	GU238159	FJ427084	KT389557	FJ427188
*A. zantedeschiae*	CBS 229.32	KT389690	KT389473	KT389558	KT389767
*A. zantedeschiae*	ICMP 16850	KY742197	KY742043	KY742131	KY742285
*Stagonosporopsis loticola*	CBS 562.81*	GU238192	GU237890	KT389684	GU237697

**Table 2.** DNA base differences amongst our strains and related species in four gene regions.

**Species**	**Strain number**	**ITS (1-494bp)**	***rpb2* (495-1090bp)**	***tub2* (1091-1424bp)**	**LSU (1425-2729bp)**
						*Allophoma thunbergiae*	GUCC 2070.7	0	0	0	0
*A. piperis*	CBS 268.93 ^*^	21	39	26	0
*A. minor*	CBS 325.82^*^	6	1	9	0
*A. pterospermicola*	GUCC 2070.3	0	0	0	0
*A. pterospermicola*	GUCC 2070.6	5	0	1	4
*A. pterospermicola*	CGMCC 3.19245^*^	1	0	1	3
*A. siamensis*	MFLU 17-2281	4	55	1	6

**Table 3.** The pycnidia, conidiogenous cells, and conidia morphology of the new species compared to known species of *Allophoma*.

**Species**	**Pycnidia**		**Conidiogenous cells**	**Conidia**		**References**
	**Shape**	**Size (μm)**	**Size (μm)**	**Shape**	**Size (μm)**	
*Allophoma alba*	(sub-)globose to ellipsoidal, whitish at onset	205−635 × 195−510	3.5−6.5 × 4.5−9	oblong, with both ends rounded, hyaline, smooth and thin-walled, aseptate	3−4.5 × 1.5−2.3	Hou et al. (2020b)
*A. anatii*	(sub-)globose to ellipsoidal	130−400 × 120−370	5−7 × 5.5−9	oblong with both ends rounded or ovoid, smooth and thin-walled, hyaline, aseptate	3.5−5.5 × 2−3	Hou et al. (2020b)
*A. cylindrispora*	glabrous, ovoid	120−210 × 90−140	3.5−4 × 4.5−5	aseptate, hyaline, smooth and thin-walled, cylindrical	3−4 × 2	Valenzuela-Lopez et al. (2018)
*A. hayatii*	(sub-)globose with 1−2 narrow and long necks	125 × 102	-	oblong to ellipsoidal	3.3−8 × 2.2−3.3	Babaahmadi et al. (2018)
*A. labilis*	globose	250 × 70	5−7 × 4−8	oblong to ellipsoidal	4−6.5 × 2–3	De Gruyter and Noordeloos (1992) Boerema et al. 2004
*A. minor*	globose to broadly ellipsoidal	150−280 × 150−220	4−5.5 × 3−4.5	ellipsoidal to ovoid or slightly allantoid	3.5−4.5 × 1.8−2.5	Aveskamp et al. (2010)
							*A. nicaraguensis*	globose to flask-shaped	30−150 × 28−120	3−4.5 × 3.5−4.5	ellipsoidal to oblong	2.5−4 × 1.5−2.5	Chen et al. (2015)
*A. oligotrophica*	globose to subglobose	50−440 × 145−420	4.5−7 × 3.5−6.5	oblong to cylindrical	3−4.5 × 1.5−2.5	Chen et al. (2017)
							*A. piperis*	subglobose	115−245 × 85−230	2.5−3.5 × 2−3	ellipsoidal to ovoid or slightly allantoid	3.5−5.5 × 1.5−2.5	Chen et al. (2015)
*A. pterospermicola*	globose to subglobose, brown, glabrous	60−330 × 67−280	6−10 × 3−6	oval to oblong, occasionally bacilliform	3−5.5 × 1.5−2	Marin-Felix et al. (2019)							
							*A. siamensis*	glabrous, ovoid	70−90 × 68−85	3−6 × 4−5	hyaline, cylindrical, aseptate	3−4 × 2−3	Jayasiri et al. (2019)
*A. thunbergiae*	subglobose to irregular, dark brown	39−200 × 48−230	4.5−7 × 4−5	oblong to cylindrical, incidentally slightly obovoid	3−5 × 1.5−2.5	this study
*A. tropica*	subglobose	100−300	2−6 × 3−6	ellipsoidal	3−4 × 1−2	Boerema et al. (2004)
*A. zantedeschiae*	subglobose or depressed	90−180	-	oval or ellipsoidal	4−7 × 2.5−3.5	Boerema (1993)
